# Sustainable Mining: Reuse of Clay from Abandoned Areas in the South of Brazil for Ceramic Production Based on a Simplex Design

**DOI:** 10.3390/ma16196466

**Published:** 2023-09-28

**Authors:** Emily Saviatto, Alexandre Zaccaron, Vitor de Souza Nandi, Juliana Acordi, Sabrina Arcaro, Fabiano Raupp-Pereira, Sergio Luciano Galatto, Manuel Joaquim Ribeiro

**Affiliations:** 1 Post-Graduate Program on Materials Science and Engineering (PPGCEM), Universidade do Extremo Sul Catarinense, Avenida Universitária 1105, Universitário, Criciúma 88806-000, SC, Brazil; saviattoemily@gmail.com (E.S.); alexandrezaccaron@hotmail.com (A.Z.);; 2 Institute of Environmental and Technological Research-IPAT, Universidade do Extremo Sul Catarinense, Rod. Gov. Jorge Lacerda 3800, Sangão, Criciúma 88807-600, SC, Brazil; sga@unesc.net; 3 Materials Research and Development Center (UIDM), Polytechnic Institute of Viana do Castelo, Rua Escola Industrial e Comercial de Nun’Álvares, 4900-347 Viana do Castelo, Portugal

**Keywords:** clay ceramic, sustainable mining, ceramic blocks, simplex design

## Abstract

The environmental impact of clay mining can be minimized using extractive mineral circularity practices. Combining the available knowledge of the characteristics of different clays with statistical tools was a decisive step for the improved use of mining resources. Through blends, all the mined materials can be incorporated to produce quality ceramic products. This study identified two types of clay from abandoned mining areas in the southern state of Santa Catarina, Brazil. These raw materials were valued together with plastic clay, which is widely used in the region, to develop 10 different formulations using a mixture design method. The clays were characterized using average granulometric distribution, mineralogical composition, and chemical, thermal and plasticity analyses. The specimens were shaped by extrusion, dried in an oven, fired in a muffle furnace and characterized based on their shrinkage, water absorption and compressive strength values. Two clays with varying characteristics—one with low workability and the other with a high silica content—exhibited difficulties (generating defects) in the extrusion shaping process, which compromised the final quality of the ceramic paste. Results showed that incorporating up to 45% by mass of the low-workability clay resulted in an increase in water absorption. The more siliceous clay improved dimensional control; however, its use at high contents (~80%) decreased the mechanical resistance. Nevertheless, when used in controlled amounts, these clays can be beneficial to the production of blocks and bricks because they have the potential to improve some properties of the finished ceramic products.

## 1. Introduction

Clays are among the most abundant materials on the Earth’s surface [1,2]; thus, they are the most commonly used raw materials for construction ceramics, such as bricks and roof tiles [3,4,5,6]. Despite the introduction of newly developed materials in the construction industry, traditional clay ceramics are still widely used [7] and are considered the foundation of this sector [8]. Brazil is among the largest producers and consumers of clay ceramics [9]. Following a decline of 6.3% in 2020, the gross domestic product (GDP) of the construction sector grew by 9.7% in 2021 [10].

According to data from the National Ceramic Industry Association (*Associação Nacional da Industria Cerâmica-ANICER*) [11], Brazil has 6903 factories throughout its national territory, with most of them located in the southern and southeastern regions. The sector has an annual revenue of BRL 18 billion, representing 4.8% of the civil construction industry in the country and generating approximately 293,000 direct and 900,000 indirect jobs. The relevance of the ceramics industry at the national level is also highlighted by the fact that approximately 90% of the masonry and roofing in the country is based on clay ceramics. Furthermore, an average of 5.3 billion blocks and roof tiles are produced monthly according to the National Ceramic Industry Association [11], resulting in a monthly consumption of approximately 10.3 million tons of clay.

The Brazilian mining-ceramics sector is predominantly composed of small-scale and family-owned companies [12], often lacking the technical knowledge related to raw materials. Therefore, these ceramists only exploit the mineral portions based on an empirical evaluation of their texture [13]. This has led to the abandonment of less valuable mineral pockets, that is, clay materials with properties that are unattractive to the ceramic sector. This exploration method, which is known as ambitious mining, is an environmental concern that has been addressed in Article 48 of the Mining Code-Decree No. 227/67. This article assigns responsibility to the Federal Government for managing mineral resources, the mineral production industry, and the distribution, trade and consumption of mineral products with a specific focus on ambitious mining. Therefore, this article addresses mining conducted with a pre-established plan of noncompliance and those performed in a way that hinders the subsequent economic exploitation of the deposit [14].

Ambitious mining can result in the disposal of large volumes of clay material, leading to the abandonment of the mined area and stagnation in the environmental recovery. Thus, new areas must be explored to supply the ceramics sector. To postpone the need to obtain new mineral sources, the utilization of different raw materials must be optimized to extend the life of the deposit.

The southern region of Santa Catarina, Brazil, is a major center for clay ceramics, accounting for 2.5% of the national production [15]; however, the abandonment of areas after clay mining is a concern in the region [16]. According to the Cooperative of Mineral Exploration in the Urussanga River Basin (*Cooperativa de Exploração Mineral da Bacia do Rio Urussanga-COOPEMI*), a licensed area of approximately 120 ha is currently underutilized because of two factors: (i) their intrinsic characteristics, such as low workability and high silica concentration, and (ii) a lack of knowledge about the properties of these raw materials, which requires a significant investment for research. Therefore, efforts are being made in the Local Productive Arrangement (LPA) of the Mineral Base in Morro da Fumaça to valorize these natural resources as alternative primary mineral sources for ceramic formulations, with the aim of integrating the sector into the practices of circularity and sustainable mineral resource exploitation.

In this study, abandoned areas in the region were identified and the characteristics of the underutilized raw materials from each of these areas were determined using a statistical mixture design method. Subsequently, clay-ceramic compositions were developed based on this previous work. And finally, statistical analysis proved to be an allied tool in the development of mixtures to solve problems of poor disposal of natural resources.

## 2. Materials and Methods

### 2.1. Geological Configurations of the Study Region

**Figure 1:**
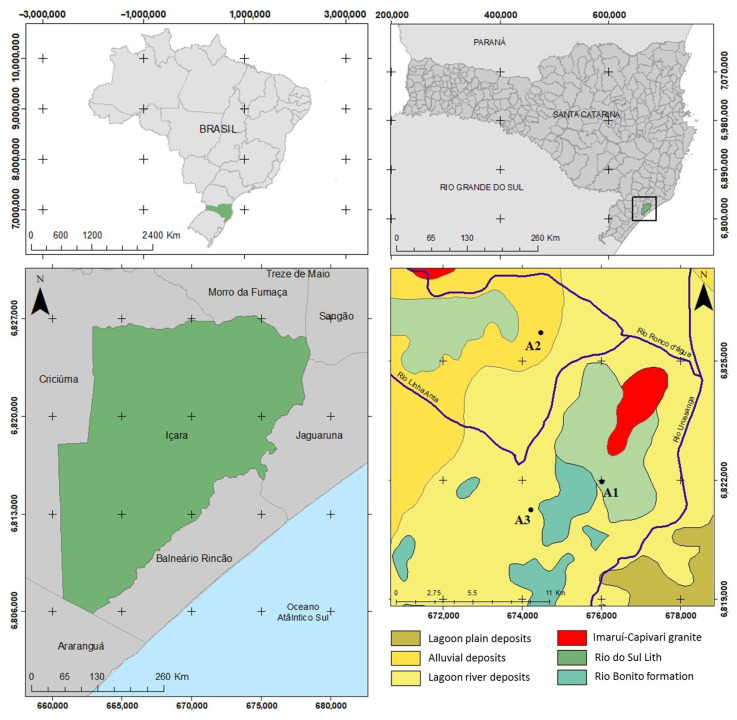
The geographical location of the study areas, referred to as A1, A2 and A3. Universal Transverse Mercator-UTM Projection. Datum Sirgas 2000. Zone 22S. Data source: IBGE (2021).

The clay ceramic production center of Morro da Fumaça is located in the southeastern region of the state of Santa Catarina; the lithological description of the production area is shown in Figure 1. In this region, sedimentary and volcanic rock outcrops form the sequence of the eastern edge of the Paraná Basin along with unconsolidated sediments from the coastal plain and current alluvial deposits. The regional crystalline basement is composed of late-post-tectonic granitoid rocks [17].

In this region, there is a geological alteration trend from the Serra Geral Mountain range to the coastal region. The Serra do Rio do Rastro can be found in the interior, where the White Column [18] is located, along with formations of the Paraná Basin—an intracratonic basin filled with sedimentary and volcanic rocks that developed between the Ordovician and Cretaceous periods. The coastal region exhibits a diversity of sand, silt and clay deposits related to both marine and continental processes, with geological representations from the Quaternary period. In the Criciúma Sheet, the outcropping sequences have ages ranging from Permian to Cretaceous [19].

### 2.2. Sample Preparation and Characterization Techniques

Three clays were used in this study: two from abandoned areas (referred to as A1 and A2), while the other (A3) was collected from a commonly used clay in the ceramic manufacturing process. Representative samples (~90 kg) of each clay were collected from different locations (Figure 1) for characterization tests.

Chemical characterization was performed using X-ray fluorescence spectrometry (Axios Max Panalytical, Malvern, UK) with wavelength dispersion (WDXRF). The loss on ignition (LoI) of the samples was performed after thermal treatment at 1000 °C, and with 3 h of dwell-time. For the characterization of the crystalline phases present in the clay samples, an X-ray diffractometer (XRD-D8 Advance Bruker, Billerica, MA, USA) was used. Data collection was conducted in the 2-theta range of 4°–72° at 40 kV and 40 mA using CuKα radiation at a wavelength of 1.5406 Å. Crystalline phases were quantified using the Rietveld method [20].

Thermal characterization was performed using differential scanning calorimetry (DSC) and thermogravimetric analysis (TGA) with a simultaneous analyzer (TA Instruments, model SDT Q600, New Castle, DE, USA). Samples were heated from room temperature to 1100 °C at a rate of 10 °C/min.

Particle size distribution was determined by laser diffractometry using the CILAS 1064 instrument between the range of 0.04 µm and 500 µm for 60 s and using sodium polyacrylate as the dispersant (Disperlan LP/G, Lamberti Brazil, Nova Odessa, Brazil). A small sample of each formulation was collected to obtain coarse particles, which are the portion of the material that cannot be disaggregated without the aid of a tool or comminution method [21]. The test involved analyzing the percentage of the materials retained on a 325 ASTM mesh sieve (45 µm) using Equation (1).

(1) $$Cp=\frac{w_{r}}{w_{0}}\times 100$$

where Cp = coarser particles (%), $w_{r}\;$ = retained weight (g) and $w_{0}$ = initial gross weight (g).

Tests were performed on each clay to measure the Atterberg plasticity index [22] through the liquid limit (LL) and plastic limit (PL) based on ISO 17892-12:2018 [23]. The samples were prepared for both tests, which consisted of dehydrating the material and passing it through a 4.8 mm sieve. The test was performed using the Casagrande method. Based on the obtained limits, the plasticity index (PI) was determined using Equation (2).

(2) PI=LL−PL

where PI = plasticity index, LL = liquid limit and PL = plastic limit.

### 2.3. Composition Development

**Figure 2:**
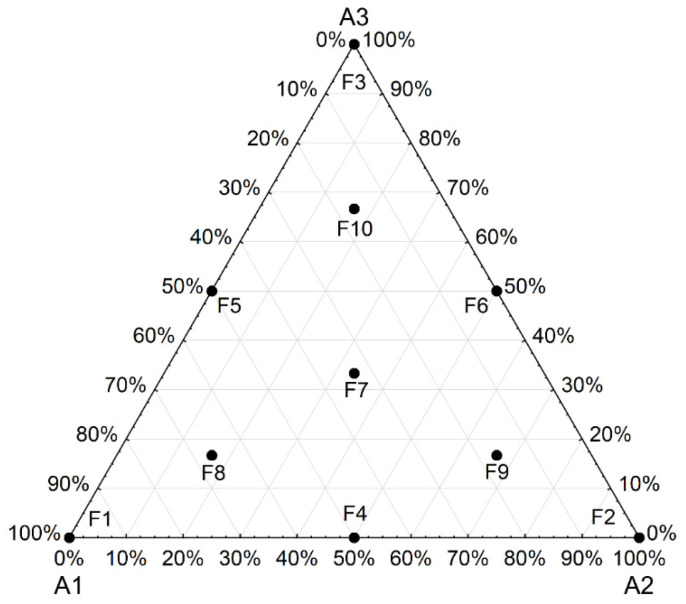
Simplex plan showing the formulations analyzed using a triaxial diagram.

The compositions were determined using the experimental design (simplex centroid), which involved defining formulations with different proportions of clays/studied materials (Figure 2). In this study, ten formulations were defined (Table 1)—three pure clays (A1, A2, and A3), two-way interactions (sides) and three-way interactions (centroids). The mixture design was based on an effective mathematical model to predict the properties of each original component, as well as their specific proportions.

The formulations were prepared on a wet basis, and for this purpose, a portion of each raw material was dried in an oven (DeLeo oven No. 2211) at 60 ± 10 °C for 24 h to determine the natural moisture content (Equation (3)).

(3) $$W=\frac{\left(w_{w}-w_{d}\right)}{w_{w}}\times 100$$

where W = water content (%), $w_{w}$ = wet weight (g) and $w_{d}$ = dry weight (g).

The water used in the study was the natural water of the raw materials. To carry out the mixtures, the water was disregarded but not removed. Approximately 10 kg (dry mass) was used for each formulation.

Subsequently, the compositions were weighed and dosed in their natural state using a scale (Marte balance, AC 10 K, precision of 0.1 g), then mixed in a laboratory-scale laminator (BERTAN equipment) and homogenized for 24 h in a sealed container.

The samples forming were determined using the vacuum extrusion method (NATREB, model NTB 140) at a vacuum pressure of 760 mmHg and a helix speed of 20 rpm. Thirty test specimens with dimensions of 36.5 × 51 × 70 mm^3^ were prepared for each formulation.

Finally, these specimens underwent thermal treatment—drying (DeLeo oven No. 2211) at 50 ± 5 °C for 24 h then at 100 ± 5 °C for an additional 24 h—to eliminate the forming moisture. All pieces were measured with a manual caliper (UNIVERSAL, precision of 0.02 mm) to determine the drying shrinkage according to Equation (4).

(4) $${Sh}_{d}=\frac{\left(L_{0}-L_{f}\right)}{L_{0}}\times 100$$

where ${Sh}_{d}$ = drying shrinkage (%), $L_{0}$ = initial length of the green pieces (mm) and $L_{f}$ = final length after drying the pieces (mm).

Firing was performed in a muffle furnace (Jung, model J200) at a heating rate of 1.7 °C/min to up to a temperature of 900 °C and with a firing dwell-time of 120 min; firing was performed in triplicate. Firing followed the traditional/representative cycle used in the ceramic industries in the Mineral LPA in the Morro da Fumaça region. All fired pieces were manually measured with calipers (UNIVERSAL, precision of 0.02 mm) to obtain their firing shrinkage according to Equation (5).

(5) $${Sh}_{f}=\frac{\left(L_{0}-L_{f}\right)}{L_{0}}\times 100$$

where ${Sh}_{f}$ = firing shrinkage (%), $L_{0}$ = the initial length after drying the pieces (mm) and $L_{f}$ = the final length after firing (mm).

After firing, a visual analysis of the pieces was conducted to identify systematic defects, such as cracks, irregular surfaces or deformations that could disqualify the product/piece.

The fired pieces were weighed (Marte AC 10 K, precision of 0.1 g) and submerged in water to determine their water absorption (WA) content. The test was conducted based on Equation (6).

(6) $$WA=\frac{\left(w_{w}-w_{d}\right)}{w_{d}}\times 100$$

where WA = water absorption (%), $w_{w}$ = weight before oven-drying (g) and $w_{d}$ = weight after oven-drying (g).

The mechanical compressive strength test was performed on 15 fired test specimens, 5 from each firing cycle. The tests were performed using a universal testing machine (EMIC DL 10000) with a force application rate of 100 N/s.

To validate the findings, the technological tests were evaluated using the analysis of variance (ANOVA) statistical technique; response surfaces were plotted to aid in their interpretation. The confidence level (probability) was set to 95%. For the analysis, the highest F-value (indicating higher significance) and lowest *p*-value (indicating higher reliability) were considered (*p* = 100 × (1 − *p*-value)). A model was selected based on its output and the adjusted coefficient of determination (R^2^). Statistical analysis was performed using the Statistica 10.0 software (Tulsa, OK, USA, StatSoft©).

After statistical analysis, the boundaries with the best results were identified to validate the study. The areas on the response surface that exhibited water absorption outside the limits established by the standard (between 8% and 25%) and mechanical strength < 1.5 MPa were disregarded. ANOVA plays a pivotal role in the fine-tuning of clay ceramic formulations by pinpointing the key factors influencing their properties. This statistical analysis allows you to improve product quality and performance.

## 3. Results and Discussion

### 3.1. Raw Materials Characterization

The chemical characterization of the studied clays (Table 2) showed that they were primarily composed of silica (SiO_2_) followed by alumina (Al_2_O_3_). The silica content of A2 (85.33%) was higher than that of the other clays. This may be associated with the high content of free silica, which is unusual in clay ceramic manufacturing. The alumina content of 7.98% may also be related to the high content of free silica, which proportionally reduces the content of other oxides and may be an indication of the presence of clay minerals. Clays with a SiO_2_ percentage > 80% have previously been studied by Zaccaron et al. [15]. Clays A1 and A3 had SiO_2_ contents of 57.57% and 69.08%, respectively, and Al_2_O_3_ contents of 22.51% and 18.20%, respectively; therefore, they were within the range of clays used in the production of ceramic materials [7,24,25,26,27].

The mass percent of alkaline and alkaline-earth oxides (CaO, MgO, K_2_O, and Na_2_O)—compounds that facilitate the sintering of ceramic materials by acting as fluxing agents [2]—in all clays was less than 1%.

Chromophore oxides (Fe_2_O_3_ and TiO_2_), which give the clay pieces reddish (F) and blue (Ti) colors after firing and are commonly used in the production of bricks and roof tiles [28,29,30,31], were also found in the clay samples. The mass percent of Fe_2_O_3_ in the A1 sample was 8.95%, representing ~10% of the chromophore oxides. In contrast, the mass percent of Fe_2_O_3_ in A2 and A3 was 2.56% and 4.45%, respectively.

The loss on ignition (LoI) is associated with the amount of chemically combined water in inorganic materials and sometimes with the presence of organic matter [32,33]. The LoI in the A2 sample (3.67%) was lower than that of the other samples, possibly owing to its high content of free silica. For clays A1 and A3, the LoI content was 9.09% and 7.20%, respectively; these values may be associated with the dehydroxylation of clay minerals and the reduction in organic matter [34,35].

**Figure 3:**
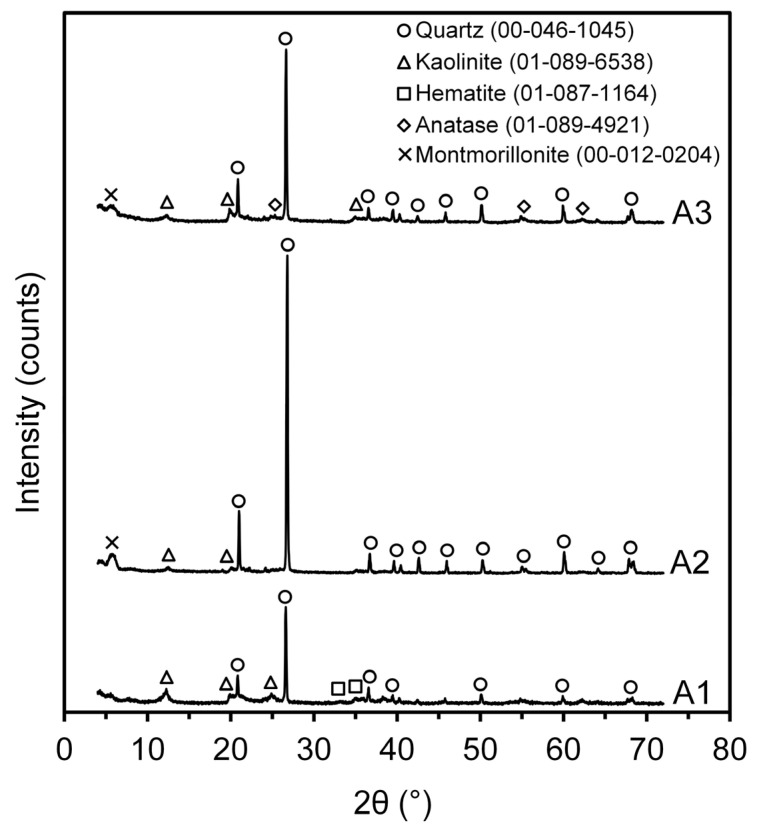
X-ray diffractogram of the studied clays.

The mineralogical compositions of the clays were determined using X-ray diffraction (XRD), as shown in Figure 3. The major crystalline phases found in the clays [36,37] were quartz (SiO_2_, JCPDS 00-046-1045) and the clay minerals, kaolinite (Al_2_(Si_2_O_5_)(OH)_4_, JCPDS 01-089-6538) and montmorillonite ((Na,Ca)_0,3_(Al,Mg)_2_Si_4_O_10_(OH)_2_.nH_2_O, JCPDS 00-012-0204). Minor minerals, such as hematite (α-Fe_2_O_3_-JCPDS 01-087-1164) and anatase (TiO_2_, JCPDS 01-089-4921), were also observed.

Quartz plays an important role in the ceramic process by acting as a deplasticizer in the extrusion process, assisting in the dimensional control of the piece, and ensuring the formation of capillaries in the shaped ceramic body, thereby facilitating drying [38,39]. Therefore, the controlled introduction of A2 into the paste can incorporate significant multifunctionalities in the manufacturing of ceramic blocks.

The characterization of kaolinitic clays with a high content of hematite (3.43% Fe_2_O_3_) is intrinsically correlated with the manifestation of a distinctive red color in the natural clay and after the firing process commonly used in the manufacturing of ceramics [40,41]. The presence of Fe^3+^ can contribute to the formation of low-melting eutectics and reduce refractoriness; additionally, anatase, which is usually associated with the TiO_2_ content, may also contribute to variations in color tone [41]. This oxide (TiO_2_) is known as a glass modifier and can act at high temperatures to form a glass phase, which has the potential to increase the strength of the samples [42]. The weight percentages of minerals based on quantitative analysis using Rietveld refinement are listed in Table 3; the quartz content of A2 (84.32%) was higher than that of the other clays, not being a content commonly used in the manufacture of clay ceramics.

**Figure 4:**
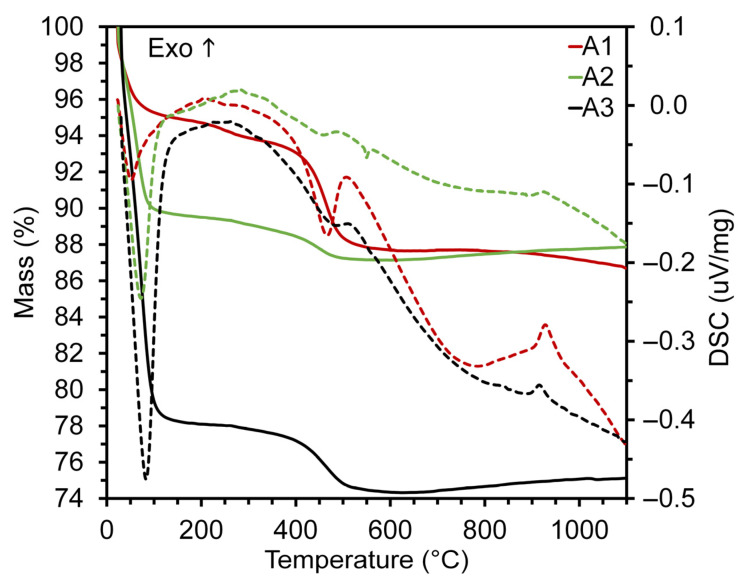
Thermal characterization of raw materials using differential scanning calorimetry (DSC) and thermogravimetric analysis (TGA). (───) Mass; (----) DSC.

The thermal behavior of the raw materials (Figure 4) showed that the initial mass loss, which occurred from room temperature (23 °C) to 200 °C, was attributed to the elimination of adsorbed water from kaolinite [43]; and in natural humidity, this accounted for a 5.3%, 10.5% and 21.9% reduction in the mass of A1, A2 and A3, respectively. Between 200 and 350 °C, there was a mass loss of <1% for all samples accompanied by a marginal exothermic inflection in the DSC curve, which was possibly related to the oxidation of organic matter.

The endothermic inflection observed in the DSC curves, Figure 4, at the temperature range of 350–650 °C was mainly due to the dehydroxylation (release of OH structural groups) of the clay minerals [44,45]. This corresponded to a mass loss of 5.94%, 1.6% and 3.12% for A1, A2 and A3, respectively (TGA curves). At 550 °C, a small endothermic peak without an associated mass loss was observed in A2, possibly related to the allotropic transformation of quartz (α-quartz to β-quartz) [46,47]. In clay ceramic manufacturing, this inversion can lead to cracks if cooling occurs abruptly [30]. Finally, the exothermic peak at 920 °C in the DSC curve corresponds to the formation of mullite [48,49,50,51].

Particle size is a fundamental aspect in the study of clays because the shape and type of mineral composition can influence the physical characteristics of the raw material. In some cases, this can affect the mechanical strength, permeability and density of the material. Finer particles can improve packing, increasing the density of the piece and its mechanical strength; however, they can also hinder drying by impeding the transfer of moisture from the interior to the surface of the piece, where the evaporation process takes place. Therefore, particle size distribution is a crucial characteristic for the processing of plastic formation and for achieving the desired properties in clay ceramic products [52].

**Figure 5:**
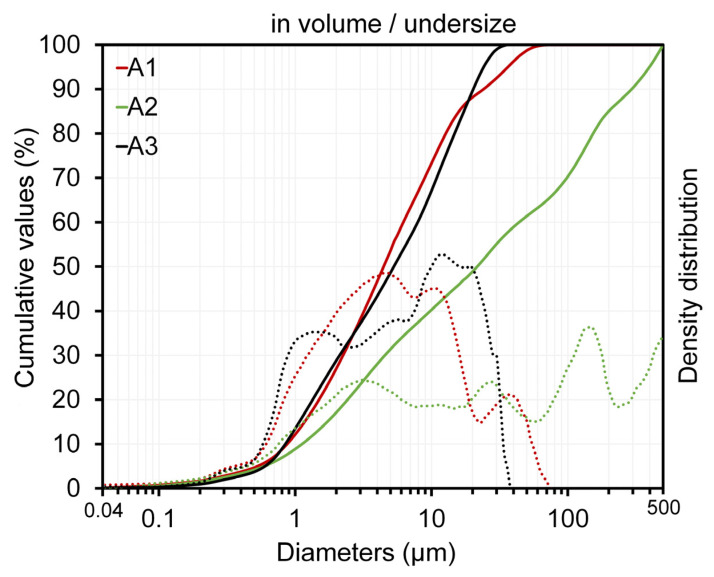
Particle-size distribution of the clays determined by laser diffraction. (—) cumulative values; (∙∙∙∙) density distribution.

The samples exhibited a multimodal particle size distribution (Figure 5). This behavior may be associated with the different morphologies and densities of the constituent particles in the clays [53,54]. The granulometric distributions of the raw materials in the fractions accumulated at 10, 50 and 90%, as well as their mean particle sizes (D_average_), are listed in Table 4.

The D_average_ of the A1 and A3 clays (8 µm) exhibited distributions with finer characteristics. However, the D_average_ of the A2 clay was >85 μm, in which some particles were >200 μm in size.

The coarse particle content retained in a particular mesh/sieve during manufacturing may be associated with the product quality, dimensional variation, mechanical strength, water absorption, extrusion rate, drying and firing properties [55].

The coarse particle content (Table 4) showed that among the clays, clay A3 (F3 composition) had the lowest free silica percentage, while that of clay A2 (F2) was the highest; clay A1 (F1) had an intermediate content (18%). Low percentages of coarse particles may be associated with a higher number of fine particles, which tends to increase the packing factor and densification of the shaped body after the drying process. In contrast, higher coarse particle percentages create a structure that contributes to dimensional stability; however, excessive amounts can adversely affect other properties, such as plasticity for the shaping process and the mechanical strength after firing [56].

**Figure 6:**
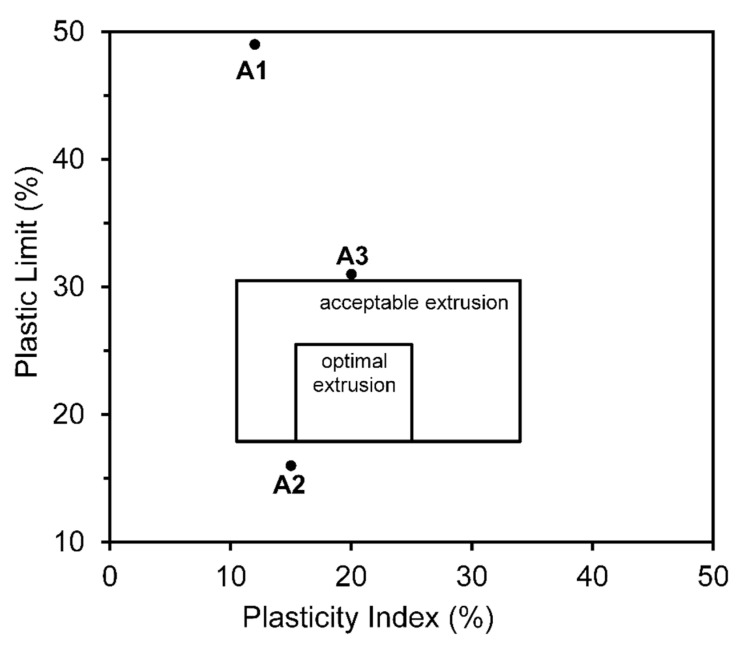
Position of studied clays on workability chart.

The Atterberg Plasticity Index (PI), which estimates the degree of clay plasticity, is presented in Table 5. According to Burmister’s classification [57] (0 nonplastic; 1–5 slight; 5–10 low; 10–20 medium; 20–40 high; >40 very high), A1 and A2 exhibited moderate plasticity (12 and 15%), while A3 was identified as a clay with good plasticity (20%). Based on these findings, a clay workability index was developed (Figure 6) using the method described by Marsigli and Dondi [58]. Despite the low D_average_ value of A1 (similar to that of A3), its plasticity was also low; this may be owing to the presence of nonplastic fine minerals, such as hematite [59], which is characteristic of a low workability index. Sample A2 was between the acceptable and optimal extrusion zones, whereas A3 was almost within the acceptable extrusion zone because of its Atterberg Plasticity Index, which was higher than that of the other clays. Therefore, clay blends can adjust these indices to provide cohesion and plasticity, which are necessary characteristics for the plastic formation of clay pieces [60]. The use of inert materials provides structural support that helps maintain the shape of the clay piece during drying and firing [61]. Therefore, the clays from the abandoned areas (A1 and A2) can be included in the ceramic formulation as more inert components and cohesion regulators, aiding in the manufacturing process of extruded clay ceramics, particularly in the drying and firing stages.

### 3.2. Technological Characterization of the Clays Compositions

**Figure 7:**
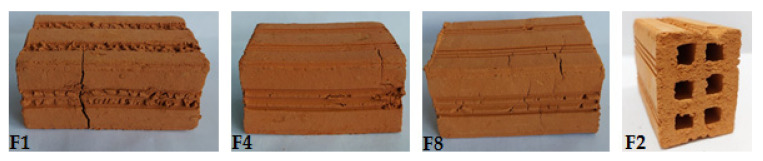
Visual analysis of parts with defects and surface roughness.

Visual analysis of the extruded ceramic blocks revealed the presence of manufacturing defects, such as cracks, irregular surfaces and deformations, which were associated with the use of clays A1 and A2. The F1, F4 and F8 compositions (Table 1), which contained levels greater than 50% of A1, exhibited cracks that occurred during extrusion (Figure 7). This was possibly owing to the lack of plasticity of clay A1. The roughness of sample F2 was also observed (Figure 7), indicating that quartz was present in excess because it increases the coarseness of the paste.

Linear thermal shrinkage is an essential technical parameter in the production of structural ceramics, as dimensional control is one of the requirements established by the standards [62].

**Figure 8:**
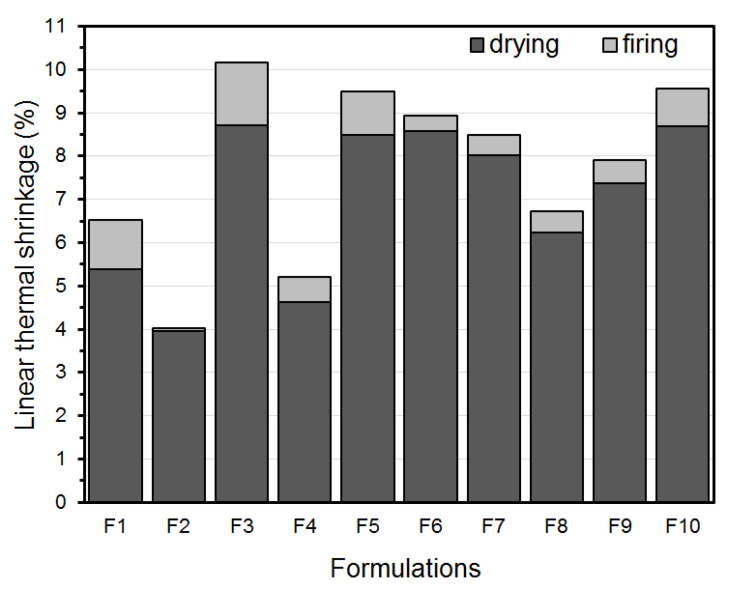
Drying and firing linear thermal shrinkage of the developed formulations processed by extrusion.

Figure 8 shows the drying and firing shrinkage behavior of the developed formulations. Sample F3, composed of a material with higher plasticity and smaller particle size, exhibited the highest drying shrinkage among the clay formulations. This is typically associated with higher densification and the high amount of water adsorbed by clay minerals. However, sample F2 had a higher gross residue content and lower drying shrinkage than the other clay formulations. This was observed because the presence of fine nonclay materials aids in water elimination and the dimensional stability of the ceramic pieces [63,64].

The firing shrinkage behavior of the formulations showed that F2, with a high content of free silica, exhibited higher dimensional stability (less shrinkage) than that of samples F1 and F3. On the other hand, the presence of fluxing oxides (higher in F1 and F3), which enhance the formation of a liquid phase in the ceramic body and the densification of the piece, may cause an increase in shrinkage [65].

According to Dondi [66], variation in drying shrinkage is “acceptable” or “optimal” between 3 and 10 cm/m and 5 and 8 cm/m, respectively. In contrast, the “acceptable” or “optimal” variations in firing shrinkage are 1.5 to 3 cm/m and <1.5 cm/m, respectively.

Clays with higher shrinkage values are more susceptible to cracks owing to the stresses generated by the shrinkage differences between the thinner and thicker areas of the piece [67]. Therefore, the characteristics of clays A1 and A2 can be used to control shrinkage during the drying process.

The technological properties—total shrinkage (Sh), water absorption (WA) and mechanical strength—of the clay formulations are presented in Table 6.

ANOVA was performed on the technological properties to validate the results (Table 7); response surfaces were plotted to aid in their interpretation. The confidence level was set at 95%. The highest F-value and lowest *p*-value, which are indicative of higher significance and reliability (*p*), respectively, were considered. The determination coefficient (R^2^) was also used to select a model.

**Figure 9:**
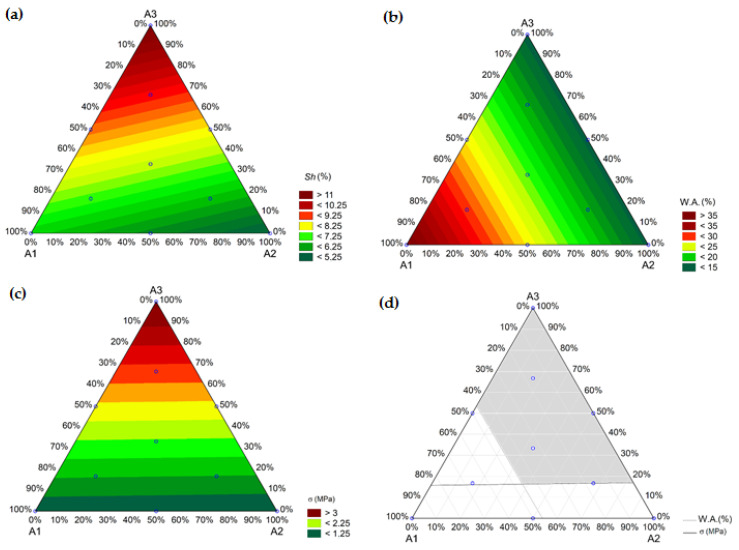
Response surface to technological characteristics—(**a**) total linear thermal shrinkage (Sh), (**b**) water absorption (WA) and (**c**) mechanical resistance in compression (σ), and (**d**) triaxial diagram illustrating the working limit for ceramic blocks.

The response surface of the total linear thermal shrinkage (Figure 9a) showed that A3 exhibited the highest total shrinkage (>11%) among the clays, while the value of A2 was the lowest (<5.25%). Regarding the water absorption (WA) test, the response surface (Figure 9b) showed that A1 was a determining factor for increasing the WA in the system; additionally, compositions with approximately 50% of this clay exceeded the 25% limit established by the technical standards [62]. In contrast, the WA of clays A2 and A3 were low, even though A2 had a high coarse particle content. This suggested that A2 may be suitable for blend development and dimensional stability. Finally, the response surface for the compressive mechanical strength test (Figure 9c) demonstrated that the inclusion of A1 and A2 clays generally decreased the mechanical strength of the pieces. In contrast, the presence of the A3 clay improved mechanical strength owing to the high densification of the ceramic bodies. Additionally, it showed that approximately 15% of A3 was required to achieve the minimum mechanical strength of 1.5 MPa, according to the technical standard [62]. Equations (7)–(9) show the fitting formulas for the selected models and the developed tests for total linear thermal shrinkage (Sh), water absorption (WA) and mechanical resistance in compression (σ), using clays one (A1), two (A2) and three (A3).

(7) Sh=6.60×A1+5.20×A2+11.16×A3

(8) WA=37.08×A1+14.58×A2+14.39×A3

(9) σ=1.06×A1+1.02×A2+3.84×A3

The working range of the studied raw materials was identified by removing the areas that exceeded 25% water absorption and that were below the 1.5 MPa compressive mechanical strength, which is in gray in Figure 9d. The incorporation of the A1 clay above ~45% significantly increased the water absorption, which exceeded the technological limits (8–25%); additionally, when the sum of the A1 and A2 clays was >80%, the compressive mechanical strength was <1.5 MPa. Therefore, the hatched area represents the applicability range of different clays based on normative specifications.

In general, conventional clays that are widely used in the production of clay ceramics are known for their favorable plasticity/workability, mechanical strength and, in porcelain products, low water absorption after firing. These traditional clays have been extensively exploited by the ceramics industry because of their availability and ease of use. The application of less conventional raw materials, characterized by high quartz content and low plasticity, represents an innovative and promising approach to the production of clay ceramics. Despite the technical challenges associated with the incorporation of less commonly used clays, such as the higher variability in their properties that require adjustments in formulations, their potential for addressing the challenging future production conditions and sustainability requirements of the sector is enormous.

The prospected exploration areas, according to the data provided by COOPEMI, contain an estimated volume ranging from 180,000 m^3^ to 220,000 m^3^, corresponding to approximately 6 ha of licensed but unexplored areas. Unique characteristics, such as a distinct mineral content (for example, A2 with 84.32% quartz) and unique chemical compositions (for example, A1 with 8.95% Fe_2_O_3_ and A2 with 93.31% SiO_2_ + Al_2_O_3_), require more process control but can be beneficial for region-specific mineral circularity actions. Production techniques, such as drying, where the A2 clay exhibits a high coarse particle content (58%) and larger grains (average diameter = 85.92 µm), may result in reduced shrinkage and dimensional stability of ceramic pieces.

The use of statistical tools has extended the lifespan of abandoned mines. The adopted evaluation approach, based on these findings, can be applied as a guide for the study of other abandoned areas. Thus, it provides dual benefits: contributing to environmental preservation through the rehabilitation of neglected areas and establishing a sustainable supply of raw materials for ceramic production within the LPA minerals, which is of great relevance to the Brazilian construction industry.

## 4. Conclusions

Through the characterization of clays from an ambitious mining area, the characteristics that explain why the mining industry does fully explore mineral resources were identified. The results showed that clay A1 exhibited low workability and A2 was characterized as a highly sandy clay owing to its high silica content (85.33%). However, using statistical tools, the viability of introducing these raw materials into ceramic matrices was determined by adjusting their formulations according to their characteristics. Clays A2 and A3 were marginally within acceptable extrusion zones; however, the forming process could be adjusted during formulation development.

In this study, the cumulative incorporation of clays A1 and A2 could be as high as 80% by mass, combined with 20% of clay A3. Using A1 in proportions exceeding 45% resulted in water absorption that exceeded the limit established by the standard (25%). The use of clay A2 improved the control of the dimensional stability of the produced ceramic pieces; however, in excess (~80%), A2 reduced the mechanical strength below the standard limit (1.5 MPa).

Finally, it was demonstrated that the development of clay blends with raw materials having different characteristics is essential for achieving a balance between ceramic product processing and the performance of sustainable production systems.

## Figures and Tables

**Table 1:** Ceramic compositions of the selected clays according to the mixture design.

Formulations	Raw Material (%)		
	Clay 1 (A1)	Clay 2 (A2)	Clay (A3)
F1	100	0	0
F2	0	100	0
F3	0	0	100
F4	50	50	0
F5	50	0	50
F6	0	50	50
F7	33.3	33.3	33.3
F8	66.6	16.7	16.7
F9	16.7	66.6	16.7
F10	16.7	16.7	66.6

**Table 2:** Chemical composition (by XRF) of clay samples.

Oxides (%)	Raw Material (Mass%)		
	A1	A2	A3
SiO_2_	57.57	85.33	69.08
Al_2_O_3_	22.51	7.98	18.20
CaO	<0.05	<0.05	0.13
Fe_2_O_3_	8.95	1.84	2.95
K_2_O	0.31	0.34	0.55
MgO	0.15	ND	0.26
MnO	0.05	<0.05	<0.05
Na_2_O	<0.05	<0.05	0.07
P_2_O_5_	0.10	<0.05	<0.05
TiO_2_	1.12	0.72	1.50
LoI	9.09	3.67	7.20

ND: not detected. LoI: loss on ignition.

**Table 3:** The content of the phases obtained from the evaluation of XRD patterns.

Clays	Phases (%)			
	Quartz	Kaolinite	Hematite	Anatase
A1	32.63	62.26	3.43	1.68
A2	84.32	15.68	-	-
A3	55.01	40.81	-	4.18

**Table 4:** Cumulative fractions (10, 50 and 90%) and average particle diameters of the clays determined using the laser diffraction technique. The coarser particles of the clays that were retained by 45 µm (325 mesh, ASTM) using a sieving technique are also shown.

Clays	Diameter (µm)			D_average_ (µm)	Coarse Particle (%)
	10%	50%	90%		
A1	0.87	4.45	24.03	8.81	18.0
A2	1.12	21.63	293.60	85.92	58.0
A3	0.84	5.29	20.23	8.13	5.0

**Table 5:** Atterberg limits of the studied clays.

Clays	LL	PL	PI
A1	61	49	12
A2	31	16	15
A3	51	31	20

LL: liquid limit; PL: plastic limit; PI: plasticity index.

**Table 6:** Results for total linear thermal shrinkage (Sh), water absorption (WA) and mechanical resistance in compression (σ).

Formulations	Sh (%)	WA (%)	σ (MPa)
F1	6.46 ± 0.84	40.66 ± 0.93	1.20 ± 0.43
F2	4.03 ± 0.38	15.59 ± 0.51	1.10 ± 0.45
F3	10.05 ± 0.61	15.46 ± 0.64	4.69 ± 1.87
F4	5.18 ± 1.09	23.81 ± 0.39	1.5 ± 0.74
F5	9.41 ± 0.55	23.73 ± 1.75	2.31 ± 0.84
F6	8.90 ± 0.54	15.46 ± 0.54	2.54 ± 0.74
F7	8.46 ± 0.55	21.30 ± 0.58	1.43 ± 0.36
F8	6.70 ± 0.39	27.53 ± 0.97	1.61 ± 0.65
F9	7.87 ± 0.54	18.44 ± 0.41	1.44 ± 0.59
F10	9.48 ± 0.69	18.20 ± 0.61	1.91 ± 0.50

**Table 7:** Analysis of variance (ANOVA) for total linear thermal shrinkage (Sh), water absorption (WA) and mechanical resistance in compression (σ).

**(** Sh **)**			
**Model**	**F**	***p***	**R^2^**
Linear	934.58	<0.001	0.73
Quadratic	71.69	<0.001	0.84
Special Cubic	3.48	0.0629	0.84
Cubic	77.44	<0.001	0.90
**(** WA **)**			
**Model**	**F**	***p***	**R^2^**
Linear	1068.30	<0.001	0.94
Quadratic	128.82	<0.001	0.98
Special Cubic	14.88	<0.001	0.98
Cubic	22.35	<0.001	0.99
**(** σ **)**			
**Model**	**F**	***p***	**R^2^**
Linear	60.10	<0.001	0.47
Quadratic	7.76	<0.001	0.55
Special Cubic	7.21	0.0081	0.57
Cubic	7.51	<0.001	0.61

F = F-value (statistical significance); *p* = *p*-value (confidence level); R^2^ = determination coefficient (adjust).

## Data Availability

Not applicable.
